# Spatio-selective activation of nuclear translocation of YAP with light directs invasion of cancer cell spheroids

**DOI:** 10.1016/j.isci.2021.102185

**Published:** 2021-02-12

**Authors:** Bernhard Illes, Adrian Fuchs, Florian Gegenfurtner, Evelyn Ploetz, Stefan Zahler, Angelika M. Vollmar, Hanna Engelke

**Affiliations:** 1 Department of Chemistry and Center for NanoScience (CeNS), Ludwig-Maximilians-Universität München, Butenandtstraße 11, 81377 Munich, Germany; 2 Department of Pharmacy, Ludwig-Maximilians-Universität München, Butenandtstraße 5, 81377 Munich, Germany; 3 Institute of Pharmaceutical Sciences, Department of Pharmaceutical Chemistry, University of Graz, Humboldtstr. 46, 8010 Graz, Austria

**Keywords:** Molecular Biology, Cell Biology, Biological Sciences Tools

## Abstract

The mechanical properties of the extracellular matrix strongly influence tumor progression and invasion. Yes-associated protein (YAP) has been shown to be a key regulator of this process translating mechanical cues from the extracellular matrix into intracellular signals. Despite its apparent role in tumor progression and metastasis, it is not clear yet, whether YAP activation can actively trigger the onset of invasion. To address this question, we designed a photo-activatable YAP (optoYAP), which allows for spatiotemporal control of its activation. The activation mechanism of optoYAP is based on optically triggered nuclear translocation of the protein. Activation of optoYAP induces downstream signaling for several hours and leads to increased proliferation in two- and three-dimensional cultures. Applied to cancer spheroids, optoYAP activation induces invasion. Site-selective activation of optoYAP in cancer spheroids strikingly directs invasion into the activated direction. Thus, nuclear translocation of YAP may be enough to trigger the onset of invasion.

## Highlights

• Photoactivation of nuclear translocation of yes-associated protein (YAP)
• Photoactivation of YAP induces downstream signaling
• Site-selective activation of YAP triggers directed invasion of cancer spheroids

## Graphical Abstract

**Figure:**
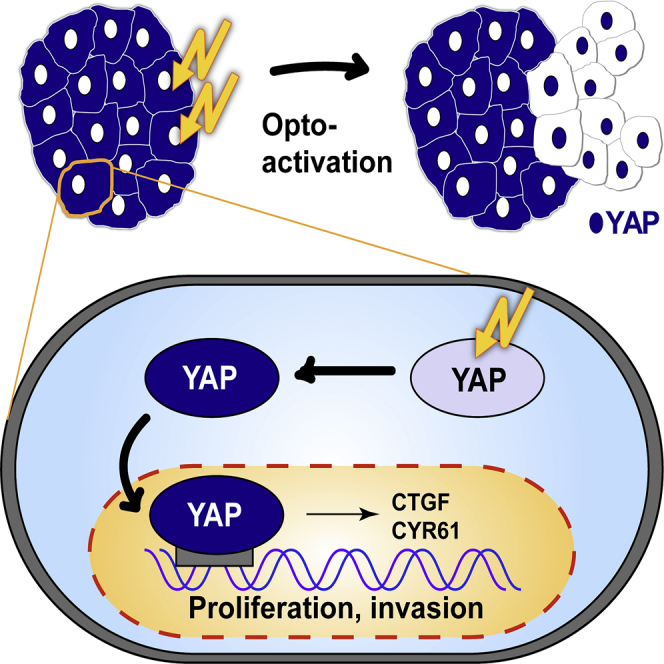

## Introduction

Yes-associated protein (YAP) is a key regulator of mechanosignaling. It translates mechanical cues from the extracellular matrix into intracellular signals (Dupont et al., 2011). This process is mediated via its localization within the cell: YAP is cytoplasmic on soft substrates and translocates into the nucleus upon sensing mechanical forces, e.g., by a stiff substrate (Dupont et al., 2011; Elosegui-Artola et al., 2017). In the nucleus, it activates downstream signaling. High nuclear YAP has been shown to be associated with invasion and altered matrix properties in tumors (Zanconato et al., 2016; Lin et al., 2015). However, due to lack of control over its translocation, it is not clear whether nuclear YAP is sufficient to induce invasion or whether it is rather just associated with invasion. YAP is able to induce symmetry breaks in cell collectives (Serra et al., 2019). We therefore hypothesized that it may be responsible for triggering the symmetry break, which is essential for the occurrence of invasive buds of a tumor and, subsequently, invasion. For a test of this hypothesis, we needed a local activation of YAP to ensure a sufficiently asymmetrical cue that induces the symmetry break. To obtain the necessary spatiotemporal control of YAP's translocation into the nucleus, we developed an optical control of YAP's nuclear translocation (Valon et al., 2017).

## Results

### Photoactivation of YAP

**Figure 1:**
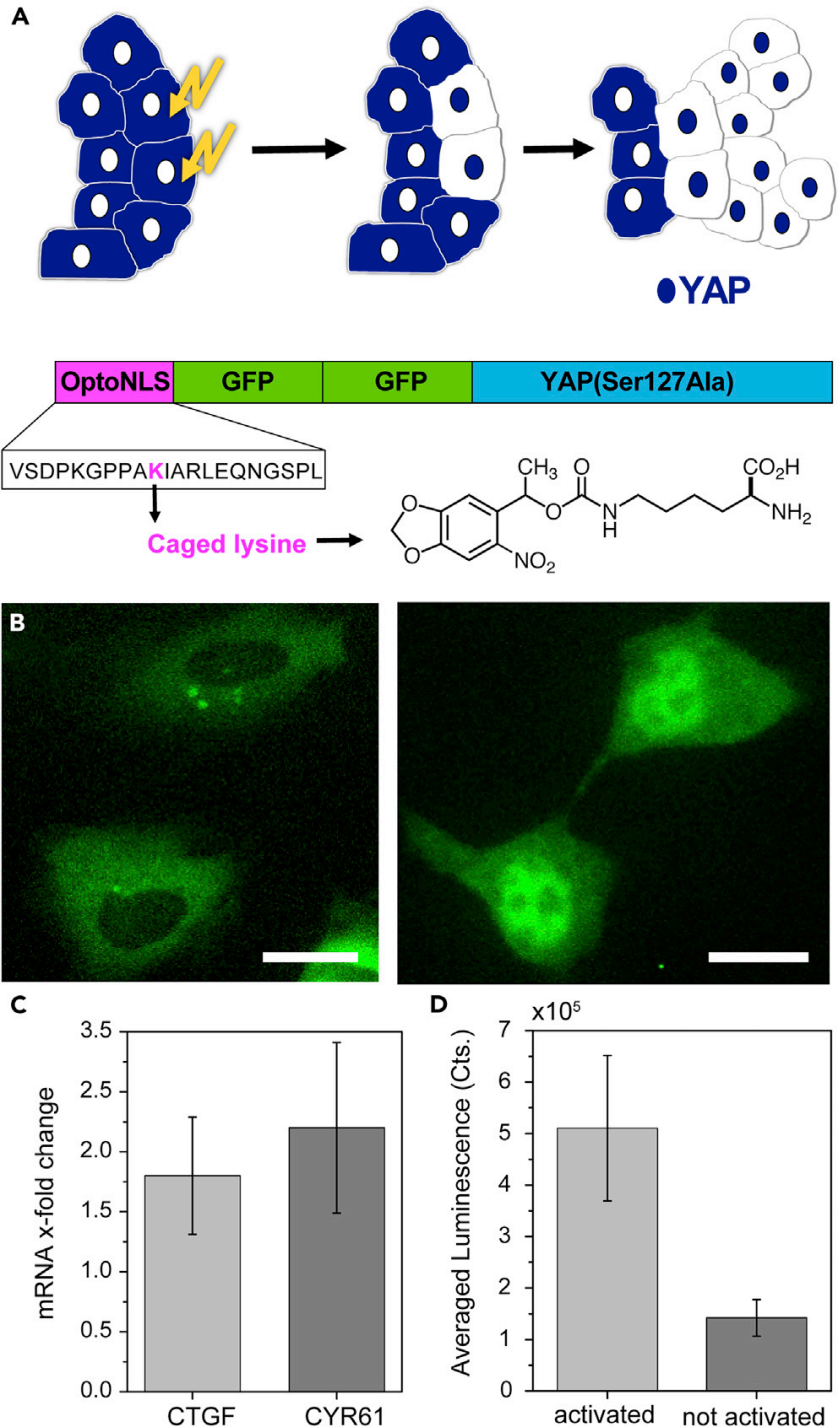
Photoactivation of YAP (A) Schematics of optoYAP. OptoYAP is composed of the optoNLS with a caged lysine and two GFP prepended to YAP Ser127Ala. The optoNLS is activated by uncaging the lysine with light leading to nuclear localization of optoYAP followed by enhanced proliferation in the photo-activated area. (B) HeLa cells transfected with optoYAP show YAP localization in the cytosol before activation. Approximately 30 min after photoactivation, optoYAP is localized in the nucleus. Scale bar = 30 μm. (C) qPCR shows an increase in RNA levels of YAP downstream proteins CTGF and CYR61 (with GAPDH as gatekeeper). (D) Yap functionality assay based on a TEAD luciferase reporter shows four times higher activity in activated samples compared to controls, which were not activated, confirming increased YAP activity. Data in (C) and (D) are represented as mean +/− standard deviation. See also related Figures S1–S7

In the cell, localization of YAP is regulated by several different processes, and the exact regulation principles are not entirely known yet (Pocaterra et al., 2020). To keep the interference with other signaling processes low and maximize the amount of control, we prepend a small (20 amino acids) photo-controlled nuclear localization signal (optoNLS) (Engelke et al., 2014), which we developed earlier, to YAP resulting in a photo-activatable YAP (optoYAP). The optoNLS is based on genetic insertion of a photocaged lysine (Gautier et al., 2010) (Fig. S1, S2) into a nuclear localization signal, which blocks nuclear import entirely (Engelke et al., 2014). Uncaging the lysine with light yields the functional signal and restores nuclear import. The small size of optoNLS and caging group minimizes their impact on protein function. To visualize protein localization and block nuclear import via diffusion by an increase in size, we use a YAP fused to two enhanced green fluorescent proteins (eGFPs). To improve YAP detection we used a stabilized form of YAP, S127A that does not affect YAP regulation by mechanical signals (Figure 1A).

Figure 1B shows images of optoYAP-transfected cells. YAP was kept cytosolic either by serum depletion or mechanically by keeping the cells on a soft matrigel substrate. Before illumination, the entire optoYAP signal is cytosolic. Less than an hour after photoactivation, most of the optoYAP signal is located in the nucleus. This successful photo-activated translocation occurred independently of whether serum depletion or matrigel was used to prime cytosolic YAP with natural signaling. After successful nuclear translocation, functionality tests were performed to show that optoYAP is fully functional and able to activate downstream signaling in the nucleus. quantitative PCR (qPCR) of CCN1 (CYR61) and CCN2 (CTGF)—proteins, which are upregulated downstream of YAP activation (Totaro et al., 2018)—reveals an enhanced signal compared to controls after photoactivation of YAP on an RNA level (Figure 1C). On a protein level, a luciferase reporter (Dupont et al., 2011) responsive to YAP signaling clearly showed enhanced expression (Figures 1D and S3) upon photoactivation of optoYAP revealing functional YAP-responsive signaling. Kinetics of YAP translocation as well as kinetics and size of downstream effects upon mechanical or chemical stimulation varies strongly depending on cell type, stimulation method, ratio of nuclear to cytosolic YAP before stimulation, and many other factors (Elosegui-Artola et al., 2017; Yu et al., 2012; Gegenfurtner et al., 2018). Kinetics of optoYAP translocation (Figures S4 and S5) and downstream effects (Figures 1C, 1D, and S3) are in the same range as measured for other stimuli (Yu et al., 2012). The absence of permanent DNA damage due to the illumination was also confirmed (Figures S6 and S7).

### Proliferation after optoYAP activation

**Figure 2:**
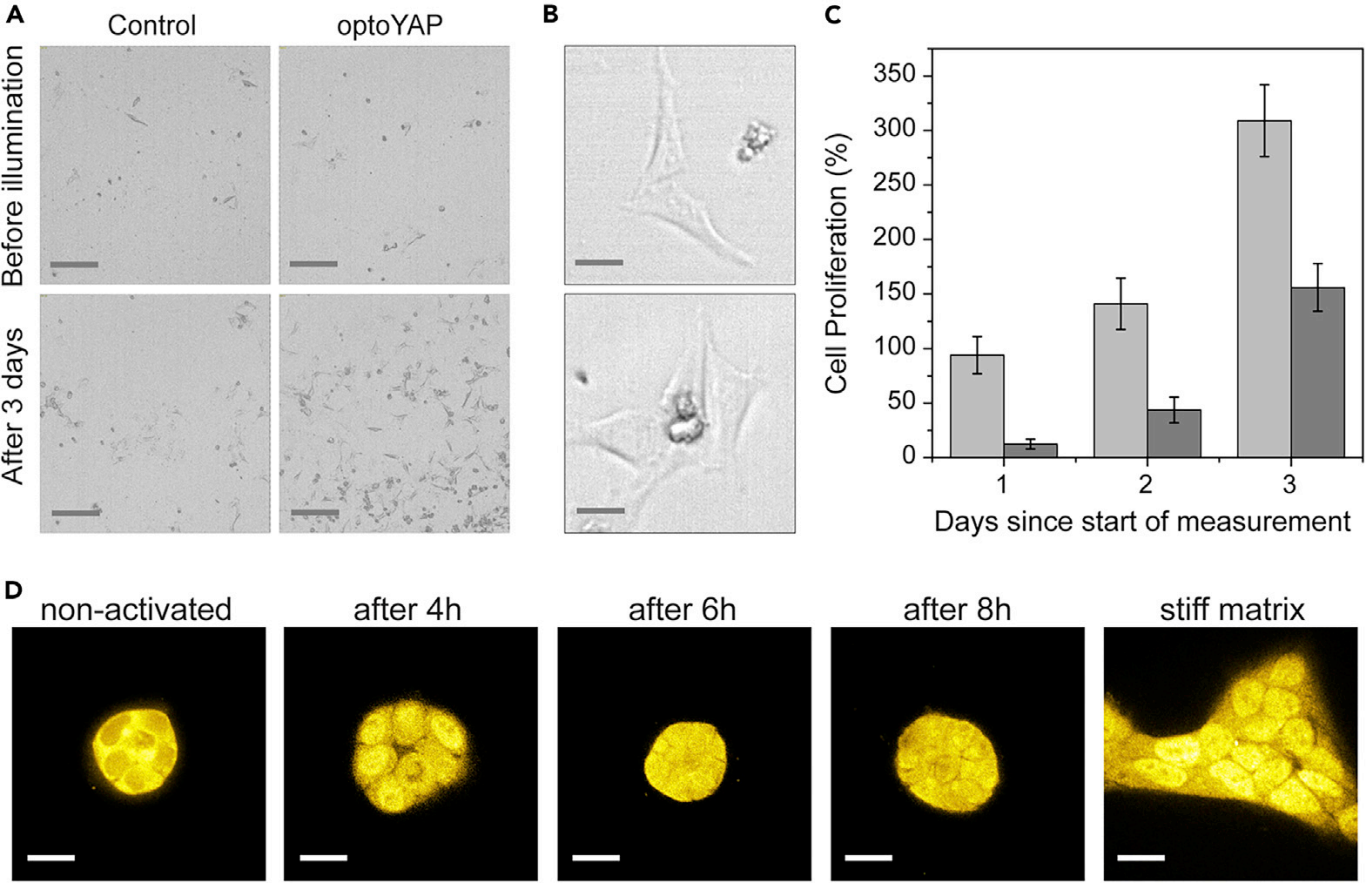
Proliferation and localization after optoYAP activation (A) Proliferation of cells with optoYAP in FBS-depleted medium is strongly enhanced by photoactivation of optoYAP compared to controls without optoYAP. Scale bar: 200 μm. (B) Higher magnification images of cells in FBS-depleted medium show no changes in morphology after photoactivation. Scale bar: 20 μm. (C) Quantitative analysis of proliferation reveals a strong increase in the growth rate of photo-activated cells (light gray) compared to controls without optoYAP (dark gray) during the first day. This difference is amplified in the following days albeit at similar growth rates compared to the control. Note that growth rates on day 1 with activated optoYAP reach levels as known for proliferation under commonly used cell culture conditions (stiff substrate, medium supplemented with serum), which generally exhibit activated YAP. Data are represented as mean +/− standard deviation. (D) Antibody staining of optoYAP transfected HeLa cells on matrigel before and after photoactivation. YAP is mainly cytosolic before activation; 4 h after activation it is located mainly in the nucleus, and at later time points, it returns to mainly cytosolic localization. For comparison, the nuclear localization of YAP in cells on a stiff matrix is shown. Scale bar: 20 μm. See also related Figures S7–S12

Having thus established a functional, photo-activatable YAP, we next studied the influence of YAP activation on HeLa cells cultured on two-dimensional substrates (Figures 2A and S8). Inactivation of YAP via serum depletion on plastic substrates did not change cell morphology. Accordingly, photoactivation of optoYAP did not lead to changes in cell morphology either (Figure 2B). However, proliferation was significantly increased: while the number of control cells without YAP activation increased after 24 h by 12.5% only, the number of cells after photoactivation of optoYAP was enhanced much stronger by 94% (Figure 2C). Note that cells with photo-activated YAP reach proliferation levels known for standard culture conditions on a plastic substrate in a medium supplemented with serum, which induce active YAP. Cells with inactivated YAP proliferate much slower. Mechanical inactivation of YAP via growth on a soft matrigel substrate leads to a round cell morphology. Upon photoactivation of YAP, interestingly, cells did not change morphology to the stretched shape found on stiff substrates (Figure S9). However, similarly to serum-depleted, activated cells, they showed increased proliferation compared to controls resulting in enhanced growth of cell spheroids on the matrigel (Figure S10). Thus, the different cell morphologies on stiff and soft substrates, respectively, do not stem from the difference in YAP activation. The results rather suggest them to be a direct consequence of mechanical forces or other signaling pathways.

### Kinetics of YAP localization and downstream signaling

To further understand the effect of the photo-activated YAP on cells, we investigated the time course of proliferation. Figure 2C already suggests the strongest effect of YAP to happen during the first day, which is then amplified by the exponential growth. A logarithmic plot and analysis (Figure S11) confirm that the growth rate of activated cells is enhanced during the first day and returns to non-activated levels on day 2 and thereafter. On a molecular level, we find an increased amount of nuclear YAP after activation. While nuclear YAP can be clearly seen 4 h after activation, the amount of nuclear YAP is decreased after 6 h (Figures 2D and S12). Finally-8 h after activation it is almost back to cytosolic localization as in the non-activated state. A time course of the luciferase reporter assay also shows an increase in downstream signaling reaching a maximum around 4-6 h after illumination and decreasing afterward (Figure S3A). The time evolution of YAP's localization and downstream luciferase expression combined with the fact that proliferation is enhanced for one day suggests that activation of YAP as performed with our photoactivation induces a boost in downstream events, which lasts for several hours and vanishes over the course of a day.

### YAP triggers directed growth in spheroids

**Figure 3:**
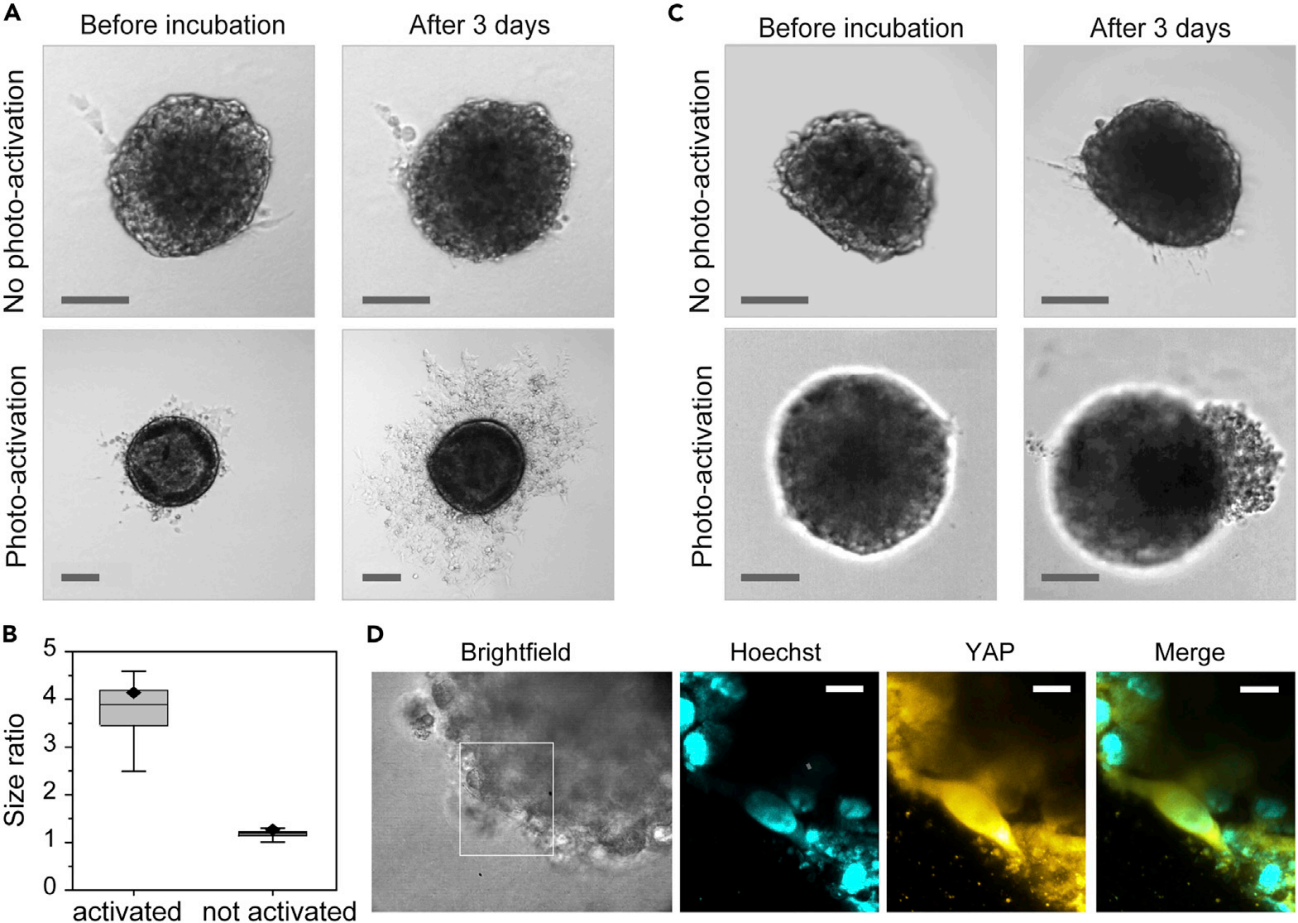
YAP triggers directed growth in spheroids (A) HeLa spheroids in collagen gels transfected with optoYAP three days after photoactivation show increased invasion leading to a larger size of the spheroid compared to non-activated controls, which barely invade at all. Scale bars: 100 μm. (B) Quantification of the average spheroid size change over three days. Activated samples (light gray) show a fourfold increase in their size (measured as area of the central plane) over three days while non-activated samples (dark gray) grew only by a factor of 1.2. (C) Spatio-selective photoactivation of spheroids with optoYAP in collagen gels on the right side of the spheroid induces directed invasion on that side, while controls, which were not photo-activated, do not show directed growth. Scale bars: 100 μm. (D) High resolution and fluorescence imaging of activated YAP in a cell in a spheroid. Scale bars: 5 μm. See also related Figures S13–S15.

Next, we investigated the effect of YAP activation on cell spheroids. To this end, spheroids were grown and embedded in collagen gels. In this three-dimensional model system, we also observed an increase in proliferation upon photoactivation of optoYAP in entire spheroids as shown in Figure 3A. Quantitative analysis (Figure 3B) reveals an increase in spheroid size by a factor of almost 4 (measured as the area of the z-projection including all connected invasive buds) over the course of three days after photoactivation of optoYAP, whereas controls hardly grew at all. Next to the enhanced size, YAP-activated spheroids also underwent morphological changes. YAP activation leads to an increased number of invading buds and network-like structures of invading cells as depicted in Figure 3A. These results already strongly suggest that activation of YAP can induce invasion.

Finally, we made use of the spatiotemporal control provided by the photoactivation. We used the spheroids in collagen gels and restricted optoYAP activation locally to a selected area of the spheroid. While non-activated controls grew symmetrically into all directions, strikingly, spatially selective activation of optoYAP was followed by invasion on the activated sites. Figure 3C shows the spherical non-illuminated spheroids and the invasive mass in the illuminated areas of photo-activated spheroids. Thus, local activation of YAP can induce a symmetry break and trigger invasion. Further microscopy studies of protein localization within the spheroids after photoactivation show the activated YAP 4 h after illumination (Figure 3D and see also Figure S13 for 3 days after illumination).

## Discussion

In conclusion, photoactivation of optoYAP in spheroids confirmed the hypothesis that YAP translocation into the nucleus is able to trigger invasion from the spheroids into the surrounding matrix. The results thus suggest YAP to be an important regulator of the onset of invasion. The developed optoYAP may also prove to be a useful tool for future investigations of the role of YAP in organ development, contact inhibition, and other processes in development and disease (Low et al., 2014).

### Limitations of the study

As a tool, optoYAP is limited to irreversible activation due to the underlying photocleavage-based mechanism. Upon brief and non-toxic photoactivation (less than 1 min), it acts as “on-switch” of a functional YAP triggering downstream signaling for several hours with spatial control as provided by the light beam used for photoactivation. The temporal control is on the order of hours (30 min until full import, 8 h until recovery to mainly cytosolic YAP), which is not as fast as triggering YAP activation by applying force directly to the nucleus (Elosegui-Artola et al., 2017), but fast enough given the timescales of downstream events and the timescales of processes in development and diseases such as the described invasion. The optoYAP construct is still sensitive to other physiological stimuli. Thus, the efficiency of photoactivation strongly depends on depletion of nuclear YAP via physiological stimuli, such as substrate stiffness or serum depletion, prior to photoactivation. On a stiff substrate in presence of serum, optoYAP will be nuclear prior to photoactivation, and thus, photoactivation will have no significant effect. Upon successful depletion of nuclear YAP prior to activation, however, it can trigger efficient changes in downstream signaling as shown above. Successful depletion and subsequent photoactivation can be monitored by fluorescence of the GFP fused to optoYAP. Although our results are limited to cell culture—specifically HeLa and A431 cells (Figures 3, S14, and S15)—the underlying method of genetic insertion of an artificial amino acid has been applied to zebrafish (Liu et al., 2017) and mice (Ernst et al., 2016). Hence, in principle, the tool should be applicable to investigations in animals as well.

### Resource availability

#### Lead contact

Further information and requests for resources and reagents should be directed to and will be fulfilled by the lead contact, Hanna Engelke (hanna.engelke@uni-graz.at).

#### Material availability

All unique reagents (optoYAP plasmid) generated in this study are available from the lead contact.

#### Data and code availability

Data and codes that support the findings of this study are available from the authors upon reasonable request.

## Methods

All methods can be found in the accompanying Transparent methods supplemental file.

## Supplemental information

Document S1. Transparent methods and Figures S1–S15
