# Supplementary material for: Spatio-selective activation of nuclear translocation of YAP with light directs invasion of cancer cell spheroids
Source: iScience. 2021 Feb 12;24(3):102185. doi: 10.1016/j.isci.2021.102185 (PMC7921841; doi:10.1016/j.isci.2021.102185)
Supplement: Document S1. Transparent methods and Figures S1–S15 [file mmc1.pdf]

iScience, Volume *24*

Supplemental information

Spatio-selective activation of nuclear translocation of YAP with light directs invasion of cancer cell spheroids

Bernhard Illes, Adrian Fuchs, Florian Gegenfurtner, Evelyn Ploetz, Stefan Zahler, Angelika M. Vollmar, and Hanna Engelke

Transparent Methods

1. Plasmid construction of optoYAP

1.1 PCR

Plasmids for optoYAP and optoYAP(Ser127Ala) were obtained as follows: GFP was subcloned into a GFP-hYAP1 plasmid on a pEGFP backbone to yield a 2xGFP-YAP fusion. Subsequently, the optoNLS from the optoNLS-TEV plasmid (Engelke et al., 2014) was prepended. Finally, the mutation Ser127Ala was introduced using the QuikChange kit (Agilent). All cloning steps except for the mutation were performed using standard protocols of the sequence and ligation independent cloning method (SLIC), which has been introduced by Li et al. (Li et al., 2007; Li et al., 2012). QuikChange was performed according to the manufacturer's manual.

The PAG plasmid for the insertion of the photocaged lysine was a gift from the Deiters lab (Engelke et al., 2014).

<u>Primers for Cloning:</u>
2xGFP-YAP:
Insert Forward ACAGGATCCCCGCATCTAGGCGCCGGCCGGATCCT
Vector Reverse: CCGGCCGGCGCCTAGATGCGGGGATCCTGTACAAT
Vector Forward: GCCAACCTGCCGGCCATGGATCCCGGGCAGCAGCC
Insert Reverse CTGCCCGGGATCCATGGCCGGCAGGTTGGCAGCGC

optoYAP:
Insert Reverse: GCCCTTGCTCACCATGCCACGGCTCTTGGTATATA
Vector Forward: ACCAAGAGCCGTGGCATGGTGAGCAAGGGCGAGGA
Insert Forward: CTACCGGTCGCCACCCCGGTCGCCACCATGGTGAG
Vector Reverse: CATGGTGGCGACCGGGGTGGCGACCGGTAGCGCTA

Primer for QuikChange to optoYAP(S127A):
Forward: TCGAGCTCATgCCTCTCCAGC
Reverse: ACATGCTGTGGAGTCAGG

1.2 OptoYAP Sequencing

Plasmid sequencing was performed by *Eurofins Genomics*.
Primers were ordered from *Metabion.*

Mutation Sequencing Primer:
GCTCTTCAACGCCGTCATGAAC

2. Synthesis and characterization of the caged Lysine

Synthesis of the caged lysine was performed as described in Gautier et al., 2010: 1-(6-Nitrobenzo[d][1,3]dioxol-5-yl)ethanol (500 mg, 2.36 mmol) and Na_2CO_3 (247 mg, 2.36 mmol) were added to THF (5 mL) and cooled to 0 °C under stirring. Next, triphosgene (701 mg, 2.36 mmol) was added to the suspension and the reaction was kept stirring overnight at RT. The reaction was centrifuged to remove Na_2CO_3 and the liquids were subsequently evaporated without heating. The residue was dried under vacuum, to yield a greyish solid (644 mg, 2.36 mmol). NMR confirms the successful synthesis of 1-(6-nitrobenzo[d][1,3]dioxol-5-yl)ethyl carbonochloridate (Fig. S1).

*N*ε-Boc-lysine (500 mg, 2.02 mmol) was dissolved in THF/1 M NaOH (aq.) (1:4 mixture, 8 mL total) under stirring and the solution was cooled to 0 °C. Next 1-(6-nitrobenzo[d][1,3]dioxol-5-yl)ethyl carbonochloridate (496 mg, 1.82 mmol) was added and the reaction was stirred overnight, at RT. The aqueous layer was washed with Et_2O (5 mL) and subsequently acidified with ice-cold 1 M HCl (20 mL) to pH 1 and then extracted with EtOAc (30 mL). The organic layer was dried over Na_2SO_4, filtered, and the volatiles were evaporated, leaving a yellow foam. The yellow foam was dissolved in DCM:TFA (1:1 mixture, 14 mL total) and the reaction was allowed to stir for 40 min. The volatiles were subsequently evaporated and the residue was redissolved in MeOH (5 mL) and precipitated into Et_2O (250 mL), yielding a white solid (679 mg, 1.42 mmol). NMR confirmed the successful synthesis of (2*S*)-2-(*tert*-Butoxycarbonylamino)-6-[1-(6-nitrobenzo[d][1,3]dioxol-5-yl)ethoxy]carbonylaminohexanoic acid which will be called caged lysine from now on (Fig. S2). For use on cells 100 mg of the caged lysine were dissolved in 1 mL H_2O and filtered with a 0.2 µm syringe filter to avoid contamination.

3. Cell Culture and optoYAP Functionality Assays

All cell experiments were prepared in a *Hera-Safe* cell culture unit from *Heraeus*. The cells were incubated in *Hera Cell incubators* also from *Heraeus*. The cells were cultured in DMEM with 10% FBS and 1% Penicillin/Streptomycin at 37°C/5% CO_2.

Experiments in which cells were not embedded in collagen gels or seeded on matrigel were performed in FBS-free DMEM to keep YAP in the cytosol prior to the photo-activation.

Experiments with cells in collagen gel or on matrigel were performed in the presence of FBS as the gels were sufficiently soft to prevent a nuclear localization of YAP even in the presence of FBS.

Cells were either seeded into *ibidi* 8-well, 6-well plates, or *Corning* 96-well plates.

Standard cell numbers for experiments were 5000 cells per well unless noted otherwise.

3.1 Transfection experiments

Transfections of cells in 8-wells plates were carried out by preparing an Optimem solution containing optoYAP plasmid (1 ug/100 µL), PAG plasmid (1 ug/100 µL) and the Xtreme Gene 9 Transfection reagent (3 µl/100 µL). After mixing carefully by tapping against the tube, the solution was then incubated at RT for 20 min. For each 8-well 10 µL of the transfection mixture were used and 3 µL of the caged Lysine were added before incubation. For the luciferase assay (1 ug/100 µL) of the 8xGTIIC-luciferase plasmid was added as well.

Transfections in 96 well plates used 5 µL of the transfection mixture and 1 µL of the caged lysine per well.

The transfection procedure for single cells and spheroids was carried out in the same manner.

3.2 Photo-activation

For non-directed photo-activation of optoYAP a *Spectroline E-Series* UV lamp (365 nm, 0.6mW/mm²) was used to illuminate the sample for 20 s. For directed photo-activation a laser (405 nm, integrated in a *Zeiss Observer SD* spinning disk confocal microscope) or LED (365 nm, integrated in a *Nikon Eclipse Ti-E*) regulated to the same output as the UV lamp was used to illuminate the sample for 20 s.

3.3 Spheroid formation

To form spheroids, 500 cells were seeded in each well (100 µL DMEM) of a 96-well plate with ultra-low adhesion and incubated at 37°C/5%CO_2 until the spheroids reached the desired size. Spheroids used for experiments had a diameter of 200-300 µm for HeLa spheroids and 100-150 µm for A431 spheroids. For transferring the spheroids into gels disposable plastic pipettes were used.

3.4 Cell Spheroid/Collagen Gel preparation

150 µL of collagen (8.36 mg/mL) were prepared in a 1.5 mL Eppendorf-tube on ice and 47.5 µL PBS and 2.5 µL 1M NaOH, both pre-cooled to 0°C on ice, were added for each well of an ibidi 8-Well plate. The reagents were mixed by pipetting up and down before applying them to the well. Aggregates were carefully aspirated with a pipette and transferred to the gels (2 per well) for a total volume of 400 µL. The spheroids were incubated for 24 h at 37°C/5%CO_2 before transfection.

3.5 Spheroid Growth Rate

To compare the growth rate of activated and inactivated optoYAP transfected spheroids, collagen embedded spheroids were imaged before activation of optoYAP and then again after three days of incubation after activation. As a control, non-activated spheroids were measured as well. The growth analysis was performed using *Fiji* by comparing 2D projections of the total area covered by spheroids and cell outgrowths before and after incubation.

3.6 RNA extraction for qPCR

RNA Extraction, purification and cDNA synthesis was carried out according to the instructions provided in the RNeasy Mini Kit from *QIAGEN.*

3.7 Luciferase Functionality Assays

HeLa cells were seeded in a 96-well plate with 5000 cells per well and then transfected with optoYAP and 8xGTIIC-luciferase plasmid. After an incubation of 24 hours, optoYAP was activated and after an additional 24 the *Bright-Glo Luciferase Assay* from *Promega* was carried out according to the instructions provided in the manual. The assays were performed on a *Berthold Tristar² LB 942*. 8xGTIIC-luciferase was a gift from Stefano Piccolo (Addgene plasmid # 34615 ; http://n2t.net/addgene:34615 ; RRID:Addgene_34615)

3.8 Proliferation Assay

500 HeLa cells were seeded into each well of a 96-well plate in media without FBS and then transfected with optoYAP after 24 hours. After an additional 24 hours, half of the wells were illuminated with UV light at 365 nm to photo-activate optoYAP. The remaining wells served as the control and were not treated with UV. Afterward, cell images were recorded every 24 hours to observe cell proliferation with and without activation of optoYAP.

4. Fluorescence imaging and staining

4.1 Confocal laser scanning microscopy

High-magnification brightfield and fluorescence microscopy was carried out utilizing a *Zeiss Observer SD* spinning disk confocal microscope with a Yokogawa CSU-X1 spinning disc unit, an oil objective with 63x magnification. For excitation, a 488 nm (GFP) and 561 nm (YAP) were used. Emission was filtered with a BP 525/50 and LP 690/50 filter, respectively. The setup was heated to 37°C and a CO_2 source was provided to keep the atmosphere at 5% for living samples. The images were recorded and processed with the *Zen* software by *Zeiss*.

4.2 High-Throughput Brightfield imaging

The proliferation assay and general spheroid growth quantification measurements were performed with the *ImageXpress Micro XLS* from *Molecular Devices* using an objective with 10x magnification and the resulting images were evaluated with the *MetaXpress* software.

Further image data analysis was performed with *ImageJ*/*Fiji* (Schindelin et al., 2012). Specific analysis methods are described at the respective assays.

4.3 High-resolution Fluorescence Confocal Microscopy

Imaging was carried out on a confocal scanning microscope (TE 300; Nikon) with mounted bright-field illumination and camera. The two-photon excitation source for Hoechst staining was a fiber-based, frequency-doubled erbium laser (FemtoFiber dichro bioMP; Toptica Photonics) running at 774 nm. The excitation laser line for YAP staining was a DPSS CW laser running at 561 nm (Cobolt Jive 50, Cobolt AB). The laser powers were 3.3 mW at 774 nm and 2.9 µW at 561, measured in front of the microscope entrance. The laser light was coupled into the microscope via a dichroic mirror (Penta Line zt405/488/561/640/785rpc; AHF Analysetechnik) that separates laser excitation and fluorescence emission. Scanning of the sample in 3D was achieved by using an xyz piezo stage (BIO3.200; PiezoConcept). The laser excitation was focused onto the sample with a 60x (water) 1.20-NA plan apochromat objective (Plan APO VC 60x 1.2 NA, Nikon). The emission was collected by the same objective and spectrally separated by a 647-nm dichroic mirror (BS 647 SP; AHF Analysentechnik). The emission was recorded with two APD detectors (Count Blue; Count Red; Laser Components) and its photons stream registered using a TCSPC card (TH260 pico dual; PicoQuant GmbH). The filter sets for the red APD were: 710/130 bandpass fluorescence filter (HQ 710/130 M; AHF Analysetechnik) and a 750 shortpass (FES0750; Thorlabs GmbH) to additionally block the 774 nm laser line. The filter sets for the blue APD were: 692/40 bandpass fluorescence filter (692/40 BrightLine HC; AHF Analysetechnik) and a 680 shortpass (HC 680/SP; AHF Analysentechnik) to additionally block the 774-nm laser line. The experiment was controlled using a home-written program written in C#. The confocal data was extracted and evaluated afterward by PAM (Schrimpf et al., 2018) and ImageJ2 (Schindelin et al., 2012).

4.4 Antibody Staining

Primary and secondary antibodies for were purchased *as stated in the following list.*

List of used antibodies:
YAP

- YAP1 polyclonal rabbit antibody; PA1-46189 *Thermo Fisher Scientific.*
- Donkey anti-Rabbit IgG (H+L) Highly Cross-Adsorbed Secondary Antibody, Alexa Fluor 546; A-10040 *Thermo Fisher Scientific.*

RPA2

- *RPA32/RPA2 mouse monoclonal antibody; ab2175, Abcam*
- *Goat anit-Mouse* IgG (H+L) Highly Cross-Adsorbed Secondary Antibody, Alexa Fluor 488; A-11001 *Thermo Fisher Scientific.*

gH2A.

- *gH2A.X rabbit antibody; 2577 Cell Signaling Technology*
- *Goat anit-Rabbit* IgG (H+L) Highly Cross-Adsorbed Secondary Antibody, Alexa Fluor 647; A-21245 *Thermo Fisher Scientific.*

4.5 2D Antibody Staining

HeLa cells were seeded on matrigel to prevent the nuclear localization of YAP and transfected with optoYAP 24h after seeding. 24 hours after transfection, cells were illuminated with UV light (365 nm) for 20 s. After another 6-24 h cells were washed with PBS (pH 7.4) before being fixed with 4% PFA for 10 min. Afterwards, cells were washed three times with PBS for 5 min each. Cells were then permeabilized with Triton X-100 (0.15% in PBS) for 10 min and washed with PBS for 5 min an additional three times. Cells were blocked with 1% BSA containing glycine for 30 min before primary antibodies were applied diluted in PBS with 1% BSA (1 µg antibody per well). After 1 h of incubation, at room temperature (or overnight at 4°C) the cells were washed 5 min with PBS three times. Next, the secondary antibodies were applied diluted in 1% BSA. After one hour of incubation the sample was washed again and Hoechst was applied before imaging.

4.6 3D Antibody Staining

HeLa spheroids embedded into collagen were fixed with 4% PFA for 40 minutes and washed with PBS twice for 20 minutes. The cells were permeabilized for 20 minutes with 0.5% Triton X-100 in PBS and subsequently washed with PBS for 30 minutes. The cells were blocked with 1% BSA in PBS overnight. Primary antibodies were diluted 1:100 with 1% BSA in PBS and cells were incubated for 72 hours. Prior to incubation with secondary antibodies (1:200 in 1% BSA), the cells were washed twice with PBS for 30 minutes. The cells were incubated with secondary antibodies for 48 hours. Afterwards, the cells were washed with PBS for 30 minutes and Hoechst 33342 (0.5 µg/ml) for 40 minutes. Prior to imaging, the cells were washed again with PBS for 30 minutes. Finally, the PBS was renewed and kept in the reservoirs during confocal microscopy.

Supplemental Figures

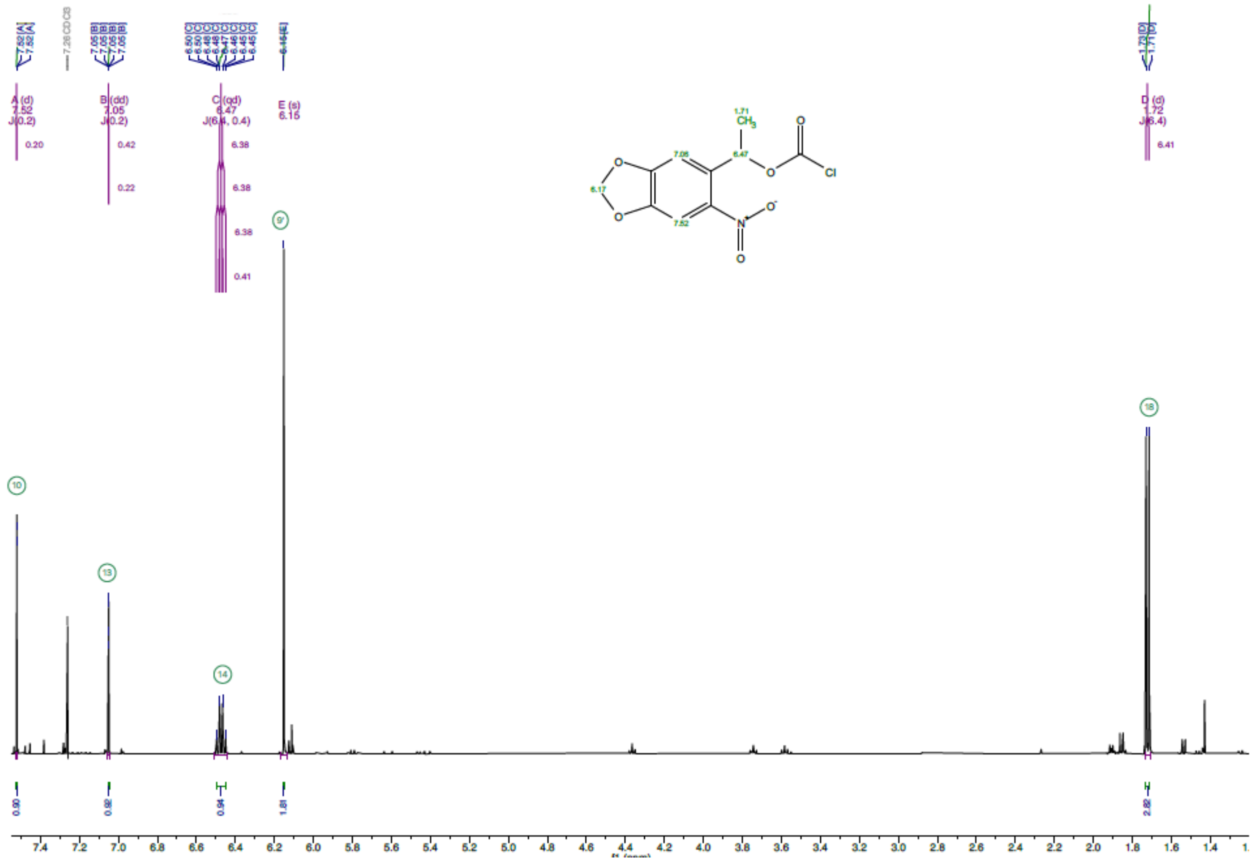

Fig. S1: NMR spectrum of 1-(6-nitrobenzo[d][1,3]dioxol-5-yl)ethyl-carbonochloridate. Related to Figure 1. It is obtained as an intermediate product of the synthesis of the caged lysine. ^{1}H NMR (400 MHz, CDCl3): _ (ppm) = 7.52 (d, J = 0.2 Hz, 1 H, H-7), 7.05 (dd, J = 0.2 Hz, 1 H, H-4), 6.47 (qd, J = 6.4, 0.4 Hz 1 H, H-1'), 6.15 (s, 2 H, H-2), 1.72 (d, J = 6.4 Hz, 3 H, H-2').

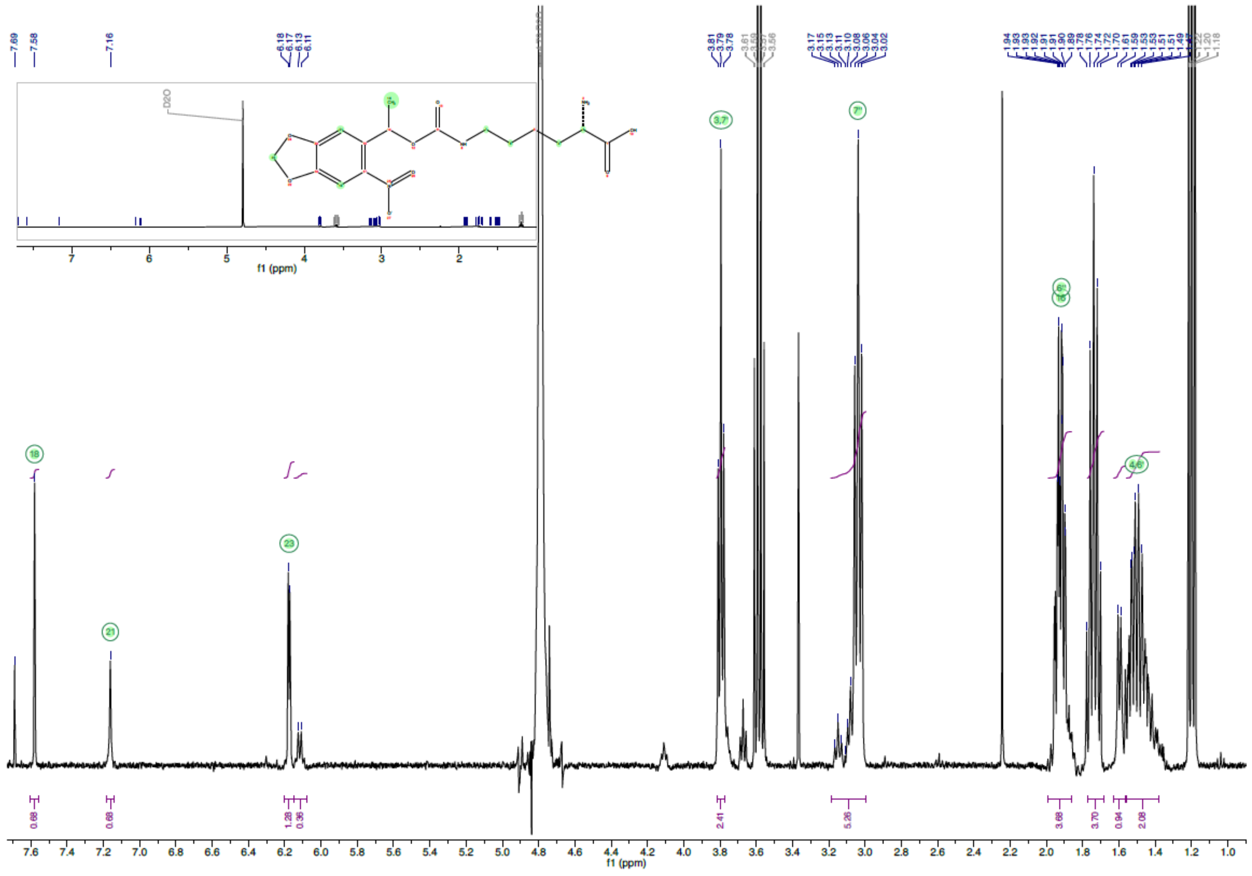

Fig. S2: NMR spectrum of the caged lysine ((2S)-2-(tert-Butoxycarbonylamino)-6-[1-(6-nitrobenzo[d][1,3]dioxol-5-yl)ethoxy]carbonyl-aminohexanoic acid). Related to Figure 1. ^{1}H NMR (400 MHz, CDCl3): _ (ppm) = 7.58 (s, 1 H, H-7"), 7.16 (s, 1 H, H-4"), 6.18 (d,J =3.5 Hz, 2 H, H-2"), 6.12 (d, J = 6.4 Hz 1 H, H-1'), 3.79 (t, J = 6.1 Hz, 2 H), 3.19 – 3.00 (m, 5 H),1.92 (m, 3 H), 1.62 – 1.38 (m, 3 H).

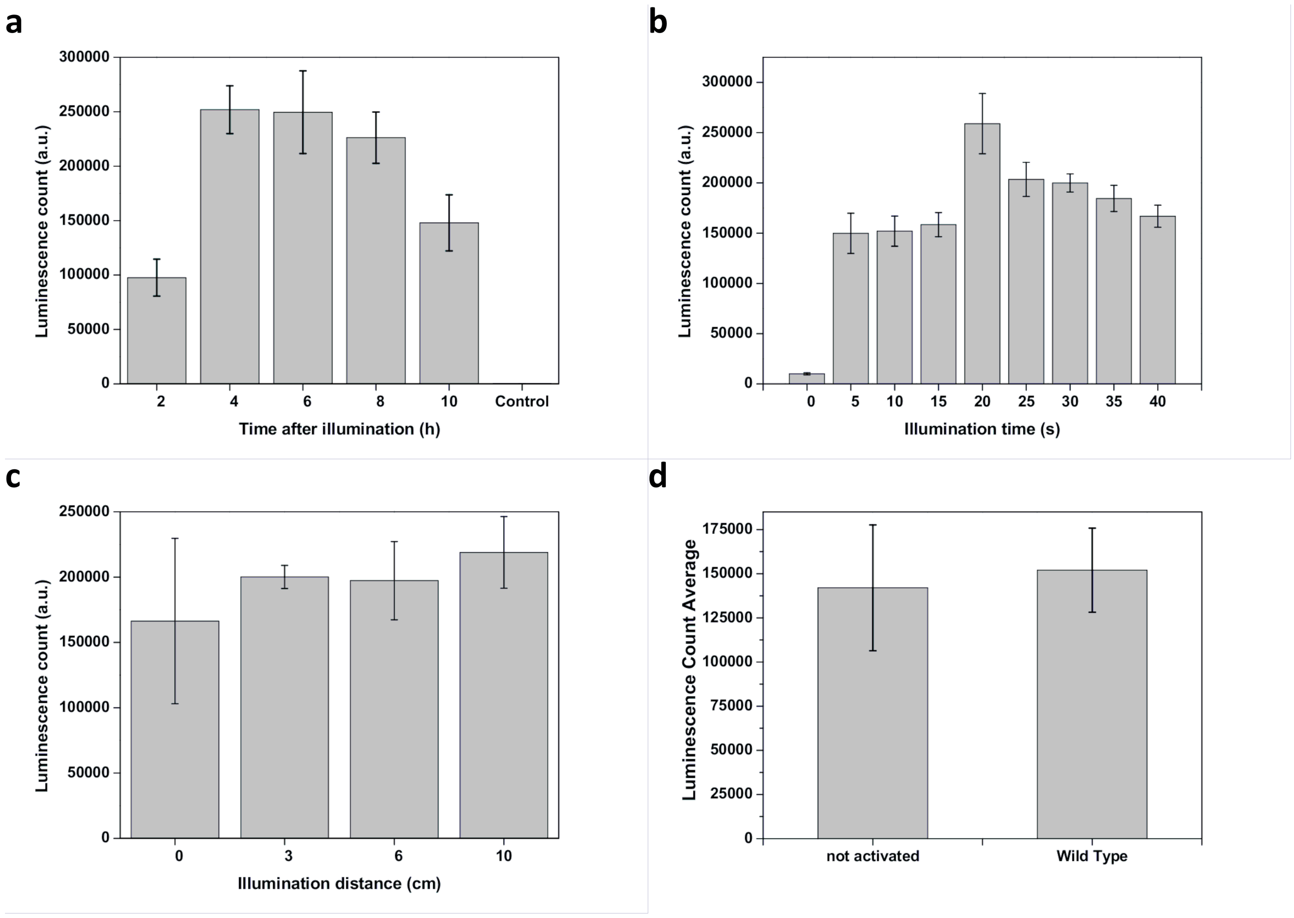

Fig. S3: Luciferase Functionality Assay. Related to Figure 1. a) Time-dependence of optoYAP activity. The observed luciferase count, and thus optoYAP activity, is time-dependent. Measurements taken at different time points after illumination show a clear maximum in activity at 4 to 6 h after photo-activation with a steady decline afterward. b) Dependence of optoYAP activity on illumination time. To gauge the effect of variations in illumination time on optoYAP activity, transfected HeLa cells were illuminated for different time periods to photo-activate optoYAP. Subsequently, the corresponding luciferase activity was recorded. The results show a clear peak in luciferase count and thus YAP activity at an illumination time of 20 seconds. c) Distance dependence of the photo-activation: The distance between light source and sample during photo-activation and thus the power needed to activate optoYAP was investigated. Photo-activation was performed at the indicated distances between light source and sample and the subsequent luciferase activity was recorded. While there was a slight deviation in recorded luminescence in case of full contact of the UV light to the sample (0 cm distance), no significant differences could be observed for larger distances. The luciferase activity shows thus little sensitivity to deviations in light intensity of the UV light source. d) Comparison of luciferase activity of optoYAP transfected, non-activated cells (not activated) and cells that were not transfected with optoYAP and not activated (Wild Type) shows that optoYAP transfection does not influence downstream signaling significantly prior to photoactivation. Data in all figures are represented as mean +/- standard deviations of triplicates.

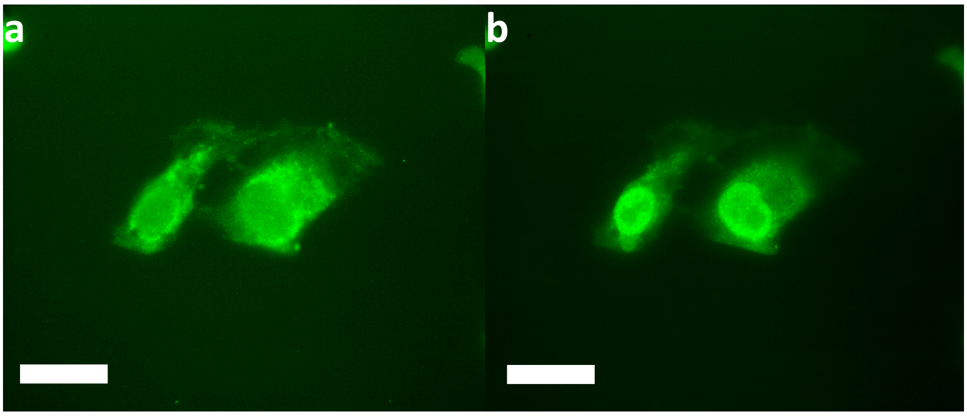

Fig. S4: optoYAP translocation. Related to Figure 1. optoYAP transfected HeLa cells on a thin layer of matrigel before (a) and 30 min (b) after illumination. Scale bar: 20 µm.

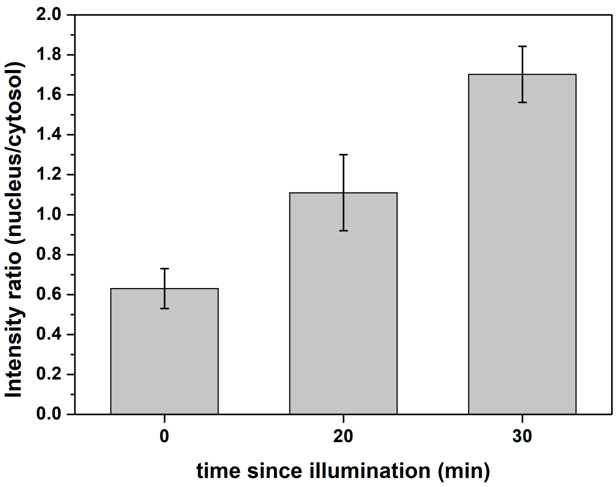

Fig. S5: Kinetics of optoYAP translocation. Related to Figure 1. Intensity ratio of (nucleus/cytosol) of optoYAP in HeLa cells in 2D cell culture at different times after optoYAP activation. Prior to activation the optoYAP signal is localized in the cytosol, but gradually translocates into the nucleus upon activation. Note: images are not background corrected for analysis, thus leading to an intensity ratio above zero before illumination, and above 1 after illumination. Data are represented as mean +/- standard deviations of triplicates.

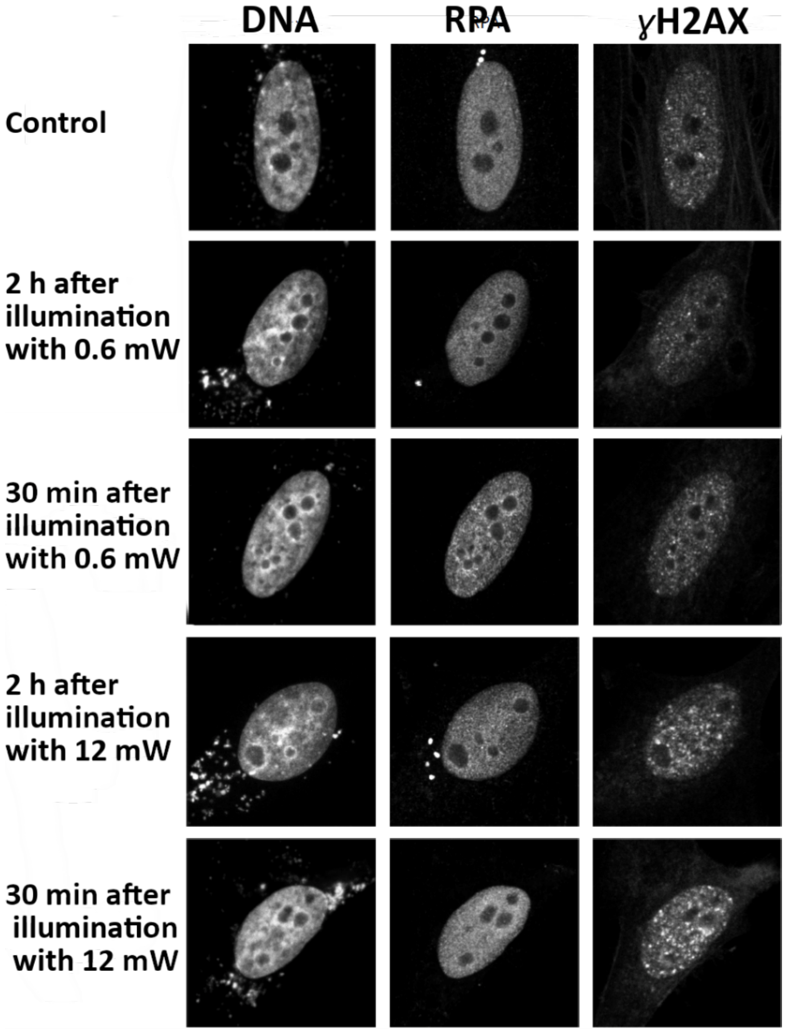

Fig. S6: Assessment of the damage caused during photo-activation with 20 s UV light (365 nm) illumination. Related to Figure 1. HeLa cells were seeded on an 8-well microscopy slide and transfected with optoYAP. After photo-activation at different intensities the damage to the cells was investigated with markers for DNA and as a marker of DNA damage with the DNA damage repair proteins RPA and γH2AX. Comparing the activated samples to a transfected non-activated control shows that the higher intensity leads to some DNA damage (visible by an increase in RPA and γH2AX signal) that is almost repaired after 2 h. The lower intensity of 0.6 mW leads to negligible DNA damage, which cannot be detected anymore after 2h. Thus, this intensity was chosen for all experiments.

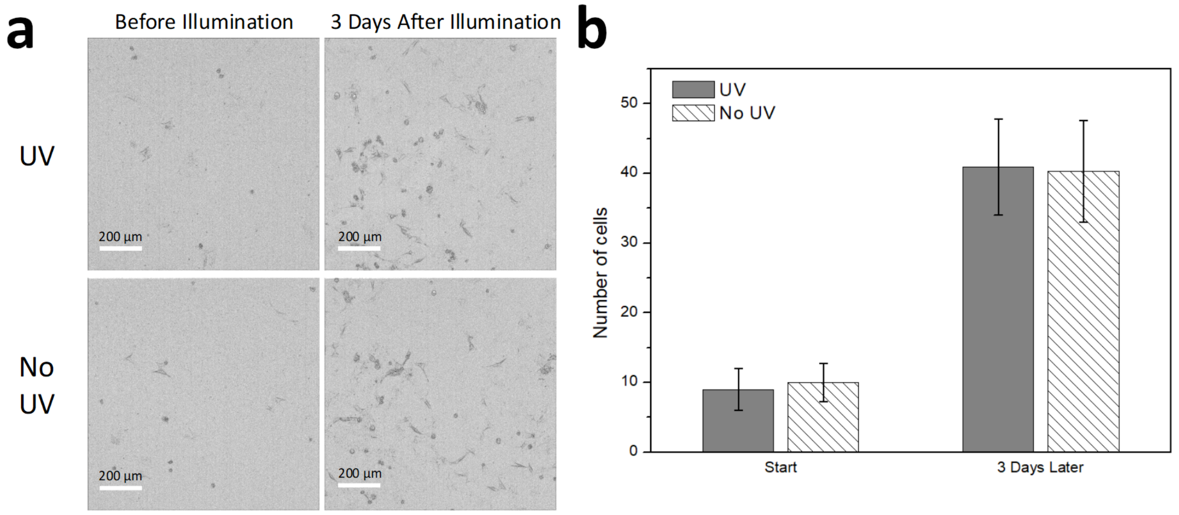

Fig. S7: Effect of UV illumination on proliferation. Related to Figure 1 and 2. Proliferation of cells is not affected by the illumination used for optoYAP activation. a) Brightfield microscopy images of cells at the time of illumination and 3 days later. The upper row was illuminated with UV as used for optoYAP activation and the lower row serves as control, which was not illuminated. Scale bar: 200 µm. b) Quantitative analysis of the number of cells at the time point of UV-illumination and 3 days later compared to controls, which were not illuminated, shows that proliferation is not influenced by the UV used for photo-activation. Data are represented as mean +/- standard deviation.

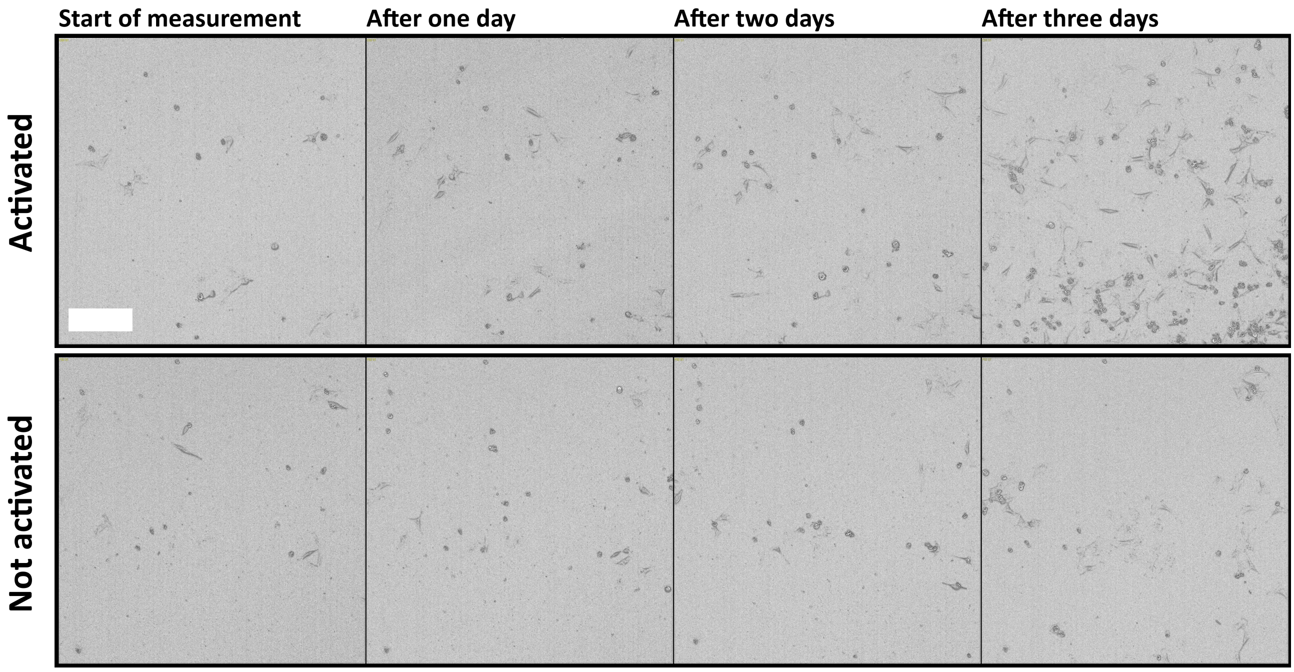

Fig. S8: Proliferation assay on optoYAP-transfected HeLa cells. Related to Figure 2. Time is measured after optoYAP activation. The upper row shows activated cells, the lower row non-activated controls. The images show a clear increase in overall growth upon optoYAP activation. Scale bar: 200 µm.

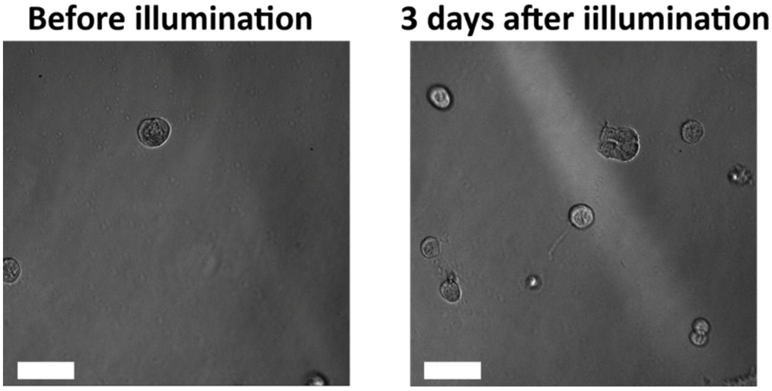

Fig. S9: Morphology of optoYAP transfected HeLa cells on matrigel. Related to Figure 2. OptoYAP transfected HeLa cells on a layer of matrigel before and three days after photo-activation. Cell morphology of transfected cells on matrigel did not change after photo-activation even after several days of incubation. Scale bar: 200 µm.

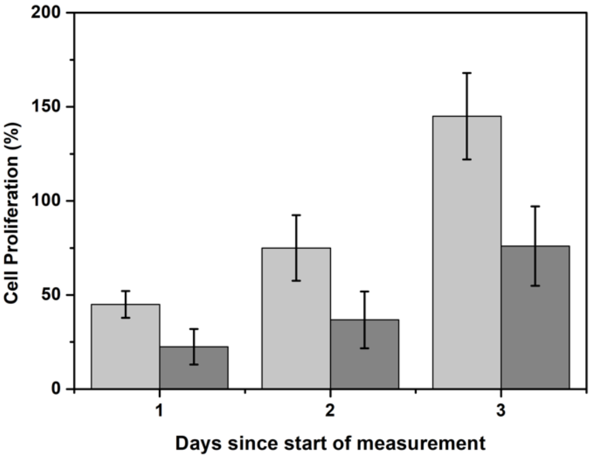

Fig. S10: Effect of optoYAP activation on proliferation on matrigel. Related to Figure 2. Cell proliferation of activated (light grey) and non-activated (dark grey) optoYAP transfected HeLa cells on matrigel in presence of FBS. The overall growth rate on matrigel is about a factor of two smaller in comparison to cell proliferation on a plastic substrate without FBS. Yet, likewise, the activated samples show a strongly increased proliferation compared to the non-activated samples. Data are represented as mean values +/- standard deviations of triplicates.

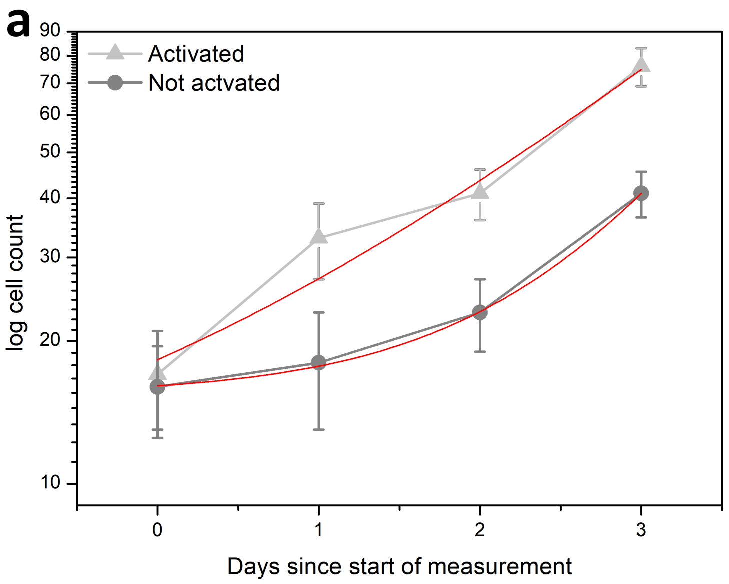

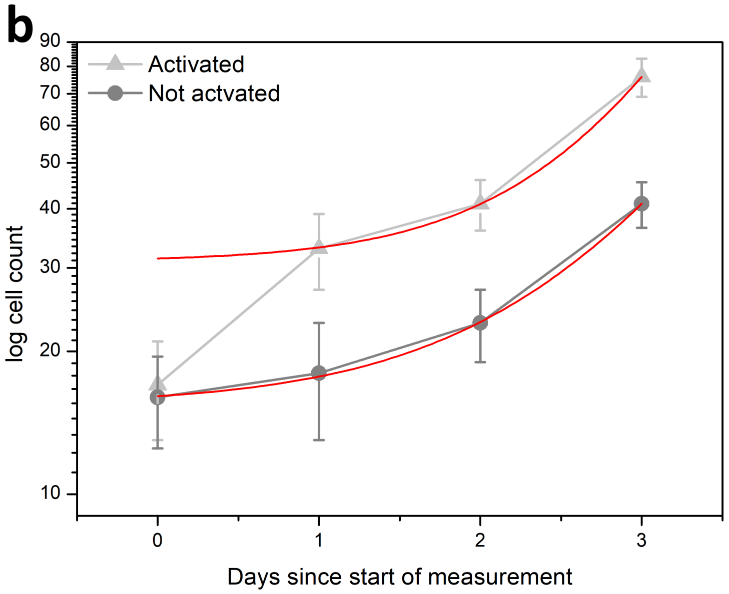

Fig. S11: Analysis of effect of optoYAP activation on proliferation. Related to Figure 2. Logarithmic plot of the cell count with exponential fits. a) Fitting exponential growth curves to the data yields an average growth rate of 0.49/d for activated and 0.31/d for non-activated cells indicating the increase in growth upon optoYAP activation. However, due to the apparent reduction in growth rate of the activated sample after day 1 the fit is only a rough approximation. b) Fitting only the last three data points of the activated sample yields a fit of much better quality and a growth rate of 0.27/d, which is very similar to that of non-activated cells. The strong deviation of the first data point from this curve shows the strongly increased growth during the first 24 h of the experiment. This leads to the conclusion that the observed difference between activated and non-activated samples mainly stems from the cell proliferation of the first day of observation, while the proliferation returns to normal growth rates after the first day. Data are represented as mean values +/- standard deviations of triplicates.

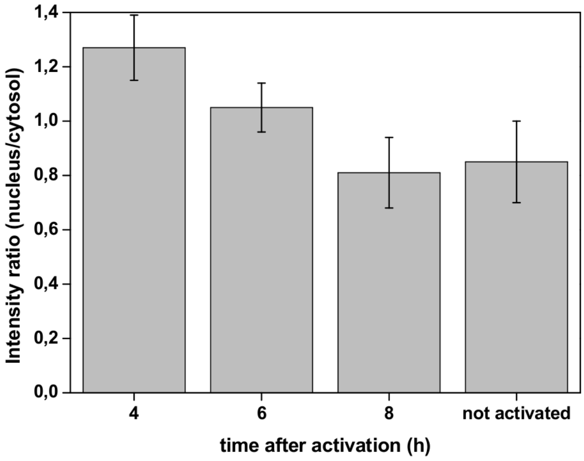

Fig. S12: Intensity ratio of (nucleus/cytosol) for YAP (via antibody staining) in HeLa cells on matrigel at different times after optoYAP activation. Related to Figure 2. 4 h after incubation YAP is in the nucleus followed by a decrease in the ratio of nuclear/cytosolic YAP. Note: images are not background corrected for analysis, thus leading to an intensity ratio above zero before illumination, and above 1 after illumination. Specifically due to the cell morphology on matrigel, the nuclear background signal is quite high. Data are represented as mean +/- standard deviations.

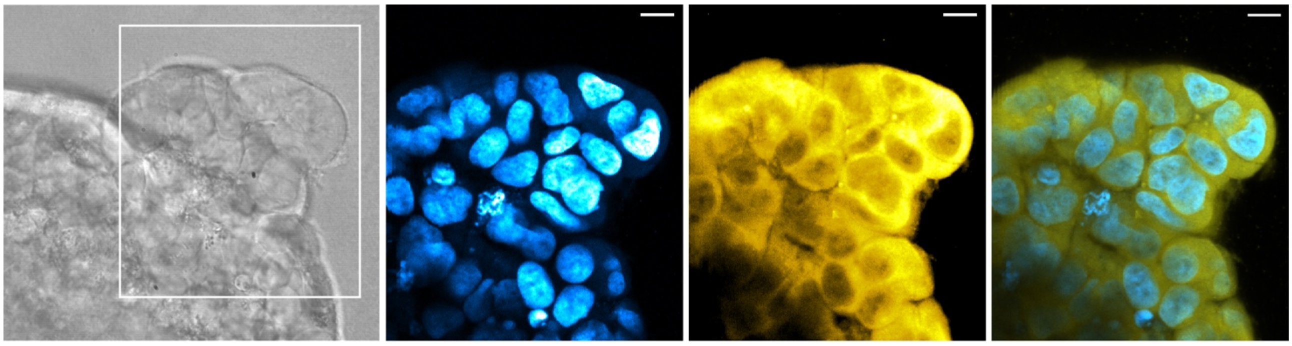

Fig. S13: High resolution confocal microscopy image of the inside of a fixed HeLa spheroid transfected with optoYAP. Related to Figure 3. The spheroid is embedded in a collagen gel and was completely illuminated for activation. The spheroid was fixed 3 days after activation. YAP appears concentrated in the cytosol. Scale bars: 5 µm.

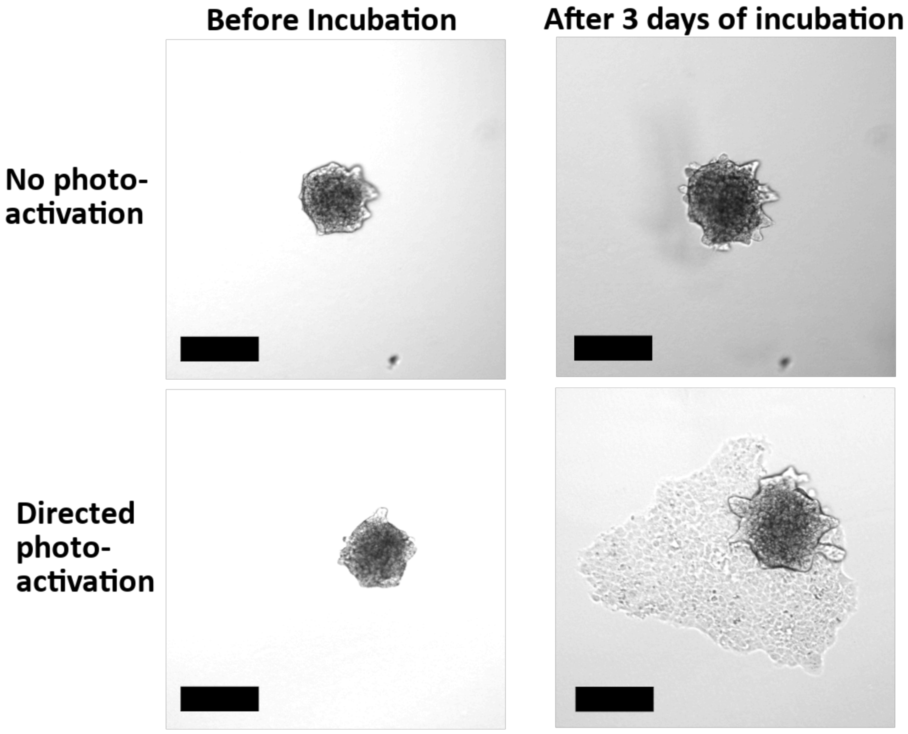

Fig. S14: optoYAP transfected A431 spheroids embedded into collagen gel. Related to Figure 3. To investigate if the effects of optoYAP activation can also be observed in other cell lines, A431 spheroids were transfected according to the same protocol described for HeLa spheroids and selectively activated. The treated A431 spheroids showed similar results to those obtained from HeLa spheroids: non-activated samples exhibit no discernible invasive behavior aside from general growth of the spheroid, while activated samples show significant invasion starting from the activated area. Scale bars: 150 µm.

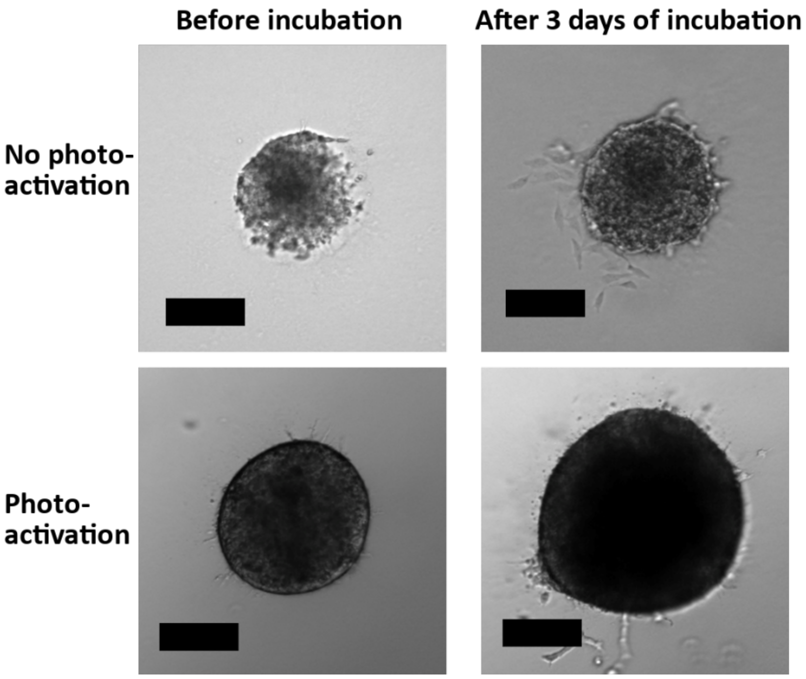

Fig. S15: Non-transfected HeLa spheroids embedded in collagen gel. Related to Figure 3. To investigate if the observed behaviors of the treated HeLa spheroids are indeed caused by optoYAP, non-transfected HeLa spheroids were used as controls and activated the same way as optoYAP. Neither the activated nor the non-activated samples show invasive behavior comparable to that of the optoYAP transfected spheroids. The only discernible difference between before and after incubation is an increase in overall spheroid size in both cases – with and without activation. Scale bars: 100 µm.

Supplemental references

Engelke, H., Chou, C., Uprety, R., Jess, P. and Deiters, A. (2014). Control of protein function through optochemical translocation. *ACS Synth Biol* 3, 731-736.

Li, M.Z. and Elledge, S.J. (2007). Harnessing homologous recombination in vitro to generate recombinant DNA via SLIC. *Nat. Methods* 4, 251-256.

Li, M.Z. and Elledge, S.J. (2012). SLIC: a method for sequence- and ligation-independent cloning. *Methods Mol. Biol.* 852, 51-59.

Schindelin, J., Arganda-Carreras, I., Frise, E., Kaynig, V., Longair, M., Pietzsch, T., Preibisch, S., Rueden, C., Saalfeld, S., Schmid, B. et al. (2012). Fiji: an open-source platform for biological-image analysis. *Nat. Methods* 9, 676-682.

Schrimpf, W., Barth, A., Hendrix, J. and Lamb, D.C. (2018). PAM: A Framework for Integrated Analysis of Imaging, Single-Molecule, and Ensemble Fluorescence Data. *Biophys. J.* 114, 1518-1528.
